# Research on Blended Teaching of Flipped Classroom Based on CNN-SSA-Bi-LSTM Deep Learning Model Computer Media

**DOI:** 10.1155/2022/3740634

**Published:** 2022-07-30

**Authors:** Feng Lu

**Affiliations:** Chang Chun Radio and TV University, Changchun 130051, Jilin, China

## Abstract

Aiming at the problem that the influencing factors of computer media flipped classroom hybrid teaching lead to the teaching effect not reaching the expected, this study proposes an ultra-short-term prediction model based on CNN-SSA-Bi-LSTM. CNN-SSA-Bi-LSTM is used to flip the study of mixed teaching in the classroom. This method constructs a one-dimensional convolutional neural network, performs data fusion and feature transformation on multiple key variables, and then constructs a two-way long-term short-term memory network prediction model, which realizes a 45-minute classroom for ultra-short-term prediction of the future. In addition, data optimization is performed through SSA to improve the predictive effect of the CNN-Bi-LSTM model. Experimental results show that compared with the traditional machine learning method, the proposed prediction model can effectively improve the prediction accuracy of the ultra-short-term classroom effect, and the relative variance of the continuous model is increased by 16.22%. High prediction accuracy and low error prove that CNN-SSA-Bi-LSTM deep learning model has strong application prospects in the research of flipped classroom hybrid teaching.

## 1. Introduction

With the rapid development of modern information technology and the arrival of the era of big data and the Internet+ [1], “University Computer Fundamentals” has become a public basic course for all majors in colleges and universities, and it is an essential part of the knowledge system for professional personnel training [2]. Its teaching goal is to comprehensively cultivate students' information literacy, cultivate students' good computational thinking ability, and enable students to apply computers to learn related courses in various disciplines, so as to improve the computer application level of college students and use computers to solve problems [3]. Blended teaching is a combination of traditional teaching methods and information technology methods [4]. The core idea of flipped classroom design is to flip the two links of knowledge acquisition and knowledge internalization in traditional teaching activities, completely changing the relationship, status, and role of teachers and students in traditional classrooms, thereby improving the quality of teaching. Therefore, it is extremely necessary to conduct research on the flipped classroom and blended teaching of university computer-based teaching.

As early as 1868, Harris, an American educator at that time, proposed a plan of “activity grouping system.” After teaching new content, teachers should provide knowledge guidance to low-level students when they are required to practice relevant skills. After the low-level students have basically mastered the knowledge system, they can explain and teach the next content [5]. From the 1930s to the end of World War II, countries were mired in economic crises and world wars and had no time to take care of the development of education. At the same time, people began to criticize and reflect on the previously hot Winnetka and Dalton systems. The hierarchical teaching experiment completely ignores the role of the teacher and the classroom and moves towards flipped teaching [6]. From the 1960s to 1979, stratified teaching in the world fell into a period of silence, most notably in the UK [7]. Since the 1960s, people have begun to pursue educational equity. Therefore, during this period, the UK underwent educational reforms, shifting from the previous structural stratification to mixed competency stratification, beginning to divide students of different abilities into one class. Middle-class students are stratified in the same class, pay attention to the differentiated development of students in the same class, and begin to move towards modern flipped teaching [8]. In 2013, the British government made changes to the standards of student achievement and progress, from selective to comprehensive, giving schools more freedom to implement blended teaching. Today, blended teaching presents more diverse characteristics, and sufficient educational resources are used to support students' all-round development of personality under blended teaching [9]. However, some researchers believe that it is difficult to achieve the expected educational effect with a single educational model, so they began to look for an optimization method, which can optimize the teaching mode according to the actual situation, so as to achieve the expected teaching effect [10]. Restricted Boltzmann Machine (RBM) is a generative network that can not only learn effective features from data but also reconstruct the original data using the learned features. In the education classroom, the results show that RBM can better predict the effect based on the original data [11]. Some studies [12] extracted the eigenvalues of classroom teaching by singular value decomposition method, and then used Bi-LSTM to effectively predict the effect of classroom teaching. Some researchers [13] proposed a Bi-LSTM-based cyclic neural network structure for the prediction of English reading classroom effects and designed a Bi-LSTM network that received random length samples for training to further improve the generalization ability of the model. Some researchers [14, 15] combined one-dimensional CNN and Bi-LSTM for aero-engine RUL prediction and used Bi-LSTM to predict the performance degradation amount in time series to obtain the future trend of performance degradation, a more accurate RUL prediction result is obtained by setting the performance degradation threshold.

## 2. Correlation Model Theory

**Figure 1:**
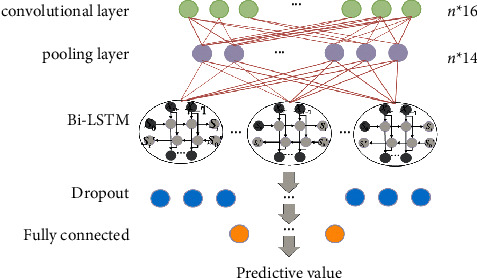
The composition of the CNN-Bi-LSTM model.

The structure of the hybrid teaching research model of the flipped classroom of computer media proposed is shown in Figure 1. It consists mainly of two parts: CNN and Bi-LSTM, in which the one-dimensional CNN network consists of a convolutional layer and a pooling layer [16–18]. The Sparrow Search Algorithm (SSA) calculates fitness values based on input values and updates data to increase prediction accuracy. The time series of various factors affect the teaching effect. Various factors are extracted and transformed through a one-dimensional CNN and then input to the Bi-LSTM layer; finally, the fully connected layer outputs the predicted effect of formic acid and media in the flipped classroom blended teaching. In addition, SSA optimization [19] is also added to the CNN data analysis.

### 2.1. Data Preprocessing and Input Variable Ordering

This study uses multisample analysis, variable selection, and data analysis from actual data, constructs a hybrid teaching prediction model for flipped classrooms, and obtains the parameters of the prediction model through training. In order to facilitate the comparison of the experimental results and ensure the reliability of the experimental data, the data in the front and rear directions are uniformly selected for prediction in this paper.

The Bayesian information criterion (BIC) [20] proposed by Schawrtz based on Bayesian theory can effectively compensate for the subjectivity of order determination of autocorrelation graphs and partial autocorrelation graphs. optimal model. Therefore, this paper chooses the BIC discriminant criterion to analyze the prediction target. The information content of the BIC criterion is defined as follows:

(1) $$\begin{array}{c} B_{\text{BIC}}=k\text{ln}\left(N\right)-2\text{ln}\left(L\right). \end{array}$$

In the formula, *k* is the number of model parameters; *N* is the number of samples; *L* is the likelihood function. For the selection of the optimal model, the maximization of the likelihood function should be considered.

According to the class requirements, the ultra-short-term prediction is a 45-minute prediction point, so the measurements of each class are collected, the average value of each 45 minutes is used as a data sample, and the BIC is used to determine the order of the models. Since the data has an obvious daily cycle, the first-order difference is performed on the 45-min time series data to make the series more stationary. Finally, four factors, such as teacher, class time, student grouping, and flipping situation, are used, and the step size is set to 6, which constitutes the input of the model, and the output of the model is the predicted value of the effect in the next 45 minutes.

### 2.2. Input Variable Fusion and Feature Extraction

As a multilayer neural network, a convolutional neural network is usually used to deal with image-related problems [21]. Continuously reduce the dimension of the image with a huge amount of data, finally, the recognition or classification of image objects is completed through several fully connected layers [22]. With the help of the two-dimensional convolutional neural network's characteristics of image feature extraction and data dimensionality reduction, this paper 1D-CNN to perform data fusion and feature on the four different types of data sequences selected above. First, the time series of the four influencing factors are used as the input of 1D-CNN, and the four classroom variables are fused through the convolution layer.

### 2.3. Bidirectional Long Short-Term Memory Network

The long short-term memory network [23] is an improvement to the RNN, which not only has the connection between the hidden layer nodes in the RNN structure but also increases the filtering of the past state. The more influential historical state is used as input. Compared with ordinary RNN models, it is more suitable for time series prediction problems, so it is more suitable for the prediction of mixed teaching in the flipped classroom [24]. As a deep learning neural network model, each neuron of LSTM is a memory cell with 3 gates, namely, the forgetting gate *f*_*t*_, input gate *i*_*t*_, and output gate *o*_*t*_. First, the forgetting gate *f*_*t*_ is multiplied by the previous cell state *C*_(*t* − 1)_ to determine which information needs to be discarded in the memory cell at the previous moment, Then, the new information is obtained by multiplying the input gate *i*_*t*_ by the alternative content *C*_*ŗt*_ that needs to be updated, and *C*_*t*_ can be obtained by discarding the information and updating the information. Finally, push the state value of *Ct* to between −1 and 1 through tan_*h*_ in the hyperbolic function, and multiply it with *o*_*t*_ to obtain the output *h*_*t*_ at the current moment. The whole process is shown in equations (2) to (7) [25], which is

(2) $$\begin{array}{c} f_{t}=\sigma \left(W_{f}\times \left[\begin{array}{cc} h_{\left(t-1\right)} & x_{t} \end{array}\right]+b_{f}\right), \end{array}$$

(3) $$\begin{array}{c} i_{t}=\sigma \left(W_{i}\times \left[\begin{array}{cc} h_{\left(t-1\right)} & x_{t} \end{array}\right]+b_{i}\right), \end{array}$$

(4) $$\begin{array}{c} {\tilde{C}}_{t}=\tan h\left(W_{C}\times \left[\begin{array}{cc} h_{\left(t-1\right)} & x_{t} \end{array}\right]+b_{C}\right), \end{array}$$

(5) $$\begin{array}{c} C_{t}=\left(f_{t}\times C_{\left(t-1\right)}\right)+\left(i_{t}\times {\tilde{C}}_{t}\right), \end{array}$$

(6) $$\begin{array}{c} o_{t}=\sigma \left(W_{o}\times \left[\begin{array}{cc} h_{\left(t-1\right)} & x_{t} \end{array}\right]+b_{o}\right), \end{array}$$

(7) $$\begin{array}{c} h_{t}=o_{t}\times \text{tan}h\left(C_{t}\right). \end{array}$$

In the formula, *h*_(*t* − 1)_ is the output at time *t* − 1; *x*_*t*_ is the input at the current time *t*; *σ* is the sigmoid function; *W* is the model weight parameter; *b* is the model bias parameter. Bi-LSTM is based on LSTM, changing the unidirectional LSTM layer into a bidirectional backward layer and forward layer. Through Bi-LSTM, classroom information in two directions is comprehensively considered, thereby improving the prediction performance of the model.

### 2.4. Sparrow Search Algorithm (SSA)

SSA [26] is a new type of intelligent optimization algorithm, and its idea originates from the foraging behavior and anticapture behavior of sparrows. The specific description of the SSA optimization process is as follows:(1) The update formula of the finder position *X*_*i*,*j*_^(*t*+1)^ is

(8) $$\begin{array}{c} X_{i,j}^{\left(t+1\right)}=\left\{\begin{array}{cc} X_{i,j}^{t}\exp\left[\frac{-i}{\alpha i_{{\text{ter}}_{\text{max}}}}\right], & R_{2}<S_{T},\\ X_{i,j}^{t}+QL, & R_{2}⩾S_{T}. \end{array}\right. \end{array}$$

In the formula, *i*_termax_ is the maximum number of iterations; *x*_*i*,*j*_ is the position of the *i*-th sparrow in the *j*-th dimension; *α* ∈ (0,1] is a random number; *R*2 ∈ [0,1] is a warning value; *ST* ∈ [0.5, 1] is a safety value; *Q* is a random number that obeys a normal distribution; *L* is a matrix of 1 × *d*, each element in the matrix is 1. (2) Joiner position *Xt*+1 *i*, *j* update

(9) $$\begin{array}{c} X_{i,j}^{\left(t+1\right)}=\left\{\begin{array}{cc} Q\exp\left[\frac{\left(X_{\text{worst}}-X_{i,j}^{t}\right)}{i^{2}},\right] & i>\left(\frac{n}{2}\right),\\ X_{p}^{\left(t+1\right)}+\left|X_{i,j}^{t}-X_{p}^{\left(t+1\right)}\right|A^{+}L, & \text{Other}. \end{array}\right. \end{array}$$

Among them, *A*^+^=*A*^*T*^(*AA*^*T*^)^−1^.

In the formula, *X*_*p*_ is the optimal position of the current finder; *X*_worst_ is the current global worst position; *A* is the matrix of 1 × *d*, in which each element is randomly assigned 1 or −1. (3) Assume that 10% ~ 20% in this group of sparrows is aware of the danger. Aware of the danger, the mathematical expression of its warning position *X*_*i*,*j*_^(*t*+1)^ is

(10) $$\begin{array}{c} X_{i,j}^{t+1}=\left\{\begin{array}{cc} X_{\text{best}}^{t}+\beta \left|X_{i,j}^{t}-X_{\text{best}}^{t}\right|, & f_{i}>f_{g},\\ X_{i,j}^{t}+K\left(\frac{\left|X_{i,j}^{t}-X_{\text{worst}}^{t}\right|}{\left(f_{i}-f_{w}\right)+\varepsilon }\right), & f_{i}=f_{g}. \end{array}\right. \end{array}$$

In the formula, *X*_best_ is the current global optimal position; *β* is the random number of the step size control parameter; *K* ∈ [−1,1] is a random number; *f*_*i*_ is the fitness value of the current sparrow individual; *f*_*g*_ is the current global optimal fitness value; *f*_*w*_ is the current global worst fitness value; *ε* is a constant [27].

The steps of the SSA are as follows:

  Step 1: initialize the population, the proportion of predators and joiners, and the number of iterations
  Step 2: calculate the fitness value, and then sort from large to small
  Step 3: update the finder location
  Step 4: update the joiner location
  Step 5: update the vigilante position (sparrow aware of danger)
  Step 6: calculate the fitness value and update the sparrow position
  Step 7: if the requirements are met, output the result; otherwise, repeat steps 2–6

### 2.5. CNN-Bi-LSTM Prediction Model

Finally, an ultra-short-term prediction model based on CNN-Bi-LSTM is established [28], whose input *X* is 4 variables, the input step size of each variable is 6, and the output *y* is the predicted value in the next 45 minutes, which is defined as follows:

(11) $$\begin{array}{c} X=\left[\begin{array}{ccc} x_{1,\left(t-6\right)} & \cdots & x_{10,\left(t-6\right)}\\ \vdots & \ddots & \vdots \\ x_{1,\left(t-1\right)} & \cdots & x_{10,\left(t-1\right)} \end{array}\right],\\ y=x_{1,t}. \end{array}$$

In the formula, *x*_1,*t*_ is the predicted value at time *t*; *x*_1,(*t* − 1)_ is the real value at the previous time (with a time interval of 45 min); *X*_*i*−4_ is the sample data of the variable.

The input *X* is input to the Bi-LSTM layer through a one-dimensional convolutional network, and the output of the Bi-LSTM layer is connected to the Dropout layer to prevent overfitting, and finally *y* is output through the fully connected layer, that is, the forecast value for the next 45 minutes.

Select classroom practice data, and obtain data samples sampled for 45 minutes according to the previous data processing method to form a training set. After many experiments, the final convolution layer number of the proposed CNN-Bi-LSTM prediction model is set to 1, the convolution kernel is set to 16, the pooling layer adopts the maximum pooling method, and the number of Bi-LSTM layers is 1, the neural network. The number of elements is 50.

## 3. Test and Result Analysis

### 3.1. Model Performance Evaluation Indicators

The evaluation indicators used in this paper include *R*, the relative mean error n*MBE*, the relative mean absolute error n*MAE,* and the relative variance n*RMSE* [29, 30]. The calculation formulas are shown in formulas (13)–(16), respectively.

(12) $$\begin{array}{c} R=\frac{\sum_{i=1}^{N}\left(I_{ci}-I_{e}\right)\left(I_{\text{mi}}-I_{m}\right)}{\sqrt{\sum_{i=1}^{N}{\left(I_{ci}-{\bar{I}}_{e}\right)}^{2}\sum_{i=1}^{N}{\left(I_{\text{mi}}-{\bar{I}}_{m}\right)}^{2}}}, \end{array}$$

(13) $$\begin{array}{c} nMBE=\frac{1}{N}\frac{\sum_{i=1}^{N}\left(I_{ci}-I_{mi}\right)}{I_{m}}\times 100\%, \end{array}$$

(14) $$\begin{array}{c} nMAE=\frac{1}{N}\frac{\sum_{i=1}^{N}\left|I_{d}-I_{\text{mi}}\right|}{I_{m}}\times 100\%, \end{array}$$

(15) $$\begin{array}{c} nRMSE=\sqrt{1/N\sum_{i=1}^{N}{\left(I_{ci}-I_{mi}\right)}^{2}/I_{m}}\times 100\%. \end{array}$$

In the formula, *I*_*ci*_ is the model predicted value; *I*_mi_ is the measured value; *N* is the overall sample size; *I*_*c*_^−^ is the average of all model predicted values; *I*_*m*_^−^ is the average of all measured values. In addition, for time series forecasting problems, continuous models are often used to measure the forecasting effect of the model. The continuous model uses the measured value at the previous moment as the predicted value at the next moment, and the prediction effect *Fs* is used to evaluate the performance of the prediction model relative to the continuous model, which is defined as follows:

(16) $$\begin{array}{c} Fs=\frac{n{\text{RMSE}}_{\text{pet}}-n{\text{RMSE}}_{f}}{n{\text{RMSE}}_{\text{pet}}}\times 100\%. \end{array}$$

In the formula, n*RMSE*_pet_ is the relative variance of the persistent model and n*RMSE*_f_ is the relative variance of the predictive model.

### 3.2. CNN-SSA-Bi-LSTM Model Performance Evaluation

First, the first group of experiments was designed; using univariate time series as input, to predict the classroom effect in the next 45 minutes, and compare the performance of LSTM and three traditional machine learning methods in ultra-short-term prediction of classroom teaching. The experimental results are shown in Table 1. The relative average error of the LSTM model is 3.04 higher than that of the other three traditional machine learning methods, and the relative average absolute error and relative variance are 14.41 and 24.34, respectively, which are smaller than the three models of SVM, BPNN, and ELM. Therefore, the ultra-short overall performance prediction of the LSTM model for flipped classroom hybrid teaching has certain advantages.

The second group analyzes the effects of the single input variable, multiple input variables, and LSTM model structure on the prediction performance. The experimental results are shown in Table 2. The single variable model uses only a single course teaching history sequence as the model input, and the multivariate model uses the four variables shown in Figure 1 as model inputs. By comparing univariate and multivariate LSTM models, it was found that adding variables that affect the teaching of the course can improve the prediction accuracy to a certain extent, especially the systematic bias (nMBE) of the predictive model. Comparing the unidirectional LSTM and bidirectional LSTM prediction models, it can be seen that adjusting the structure of the LSTM model can further improve the prediction effect. The proposed CNN-SSA-Bi-LSTM model combines CNN for data fusion and feature extraction of variables, taking advantage of Bi-LSTM's good at processing time series, the prediction performance is better than other LSTM models in Table 2.

**Figure 2:**
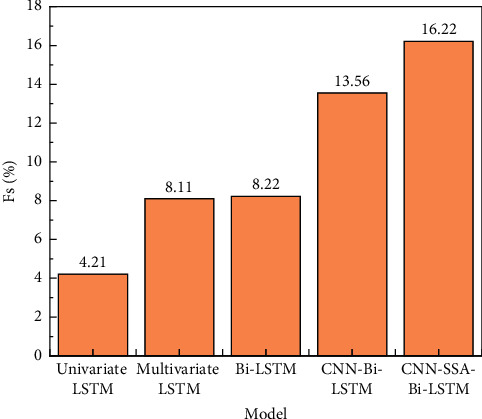
Forecast skills of different LSTM models.

Figure 2 shows the prediction effect of different LSTM models. It can be seen that the proposed CNN-Bi-LSTM improves nRMSE by about 14% compared with the continuous model, while the CNN-SSA-Bi-LSTM model reaches 16.22%, slightly higher than CNN. -Bi-LSTM model, is much higher than other prediction models. Therefore, among the five LSTM-type models used in this study, the LSTM optimized by the CNN and SSA models has higher accuracy and greater applicability.

Combining Tables 1 and 2, we can know that using multiple influencing factors as model input and constructing the CNN-SSA-Bi-LSTM model to predict classroom teaching effect, the errors nMBE, nMAE, and nRMSE are all the smallest, which are −0.18, 11.28, 20.09, respectively. The performance improves the most compared to the continuous model.

**Figure 3:**
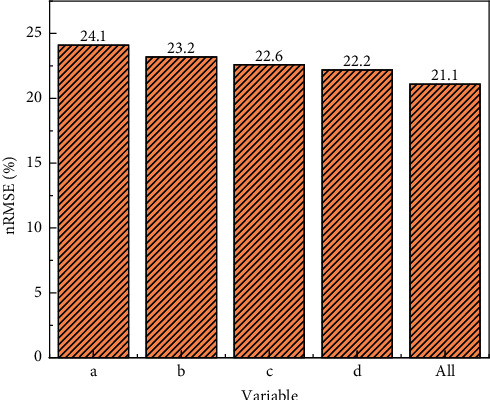
nRMSE of CNN-SSA-Bi-LSTM model without one input variable.

In addition, considering that the actual course teaching is carried out in separate batches, the 4 variables (see Figure 1) may not be obtained at the same time, to this end, the third set of experiments analyzes the impact of the absence of different input variables on the predictive performance of the proposed A model.  Figure 3 express the experimental results. Teacher, class time, student grouping, flip situation, and student grouping indicate that the missing input variable is a, and the remaining three variables on the horizontal axis are used as model input. The same as the expression a, b, c, and d indicate that the missing variables are teacher, class time, and flipping situation, respectively. All indicate that the above four variables are used as model input.

Figure 3 shows that when the student grouping is missing in the input variable, the nRMSE of the prediction result of the CNN-SSA-Bi-LSTM model is the largest, and the prediction effect is the worst, with an nRMSE of 24.1%. Therefore, among these 4 variables, student grouping is the most important for prediction 45 minutes ahead. When all the above four variables are used as model input, the nRMSE of the model prediction result is the smallest and the performance is optimal; if any one of the variables is missing, the nRMSE of the model will increase, that is, the prediction performance will deteriorate. This shows that increasing the input variables helps improve the prediction performance.

### 3.3. CNN-SSA-Bi-LSTM Model Ultra-Short-Term Prediction

**Figure 4:**
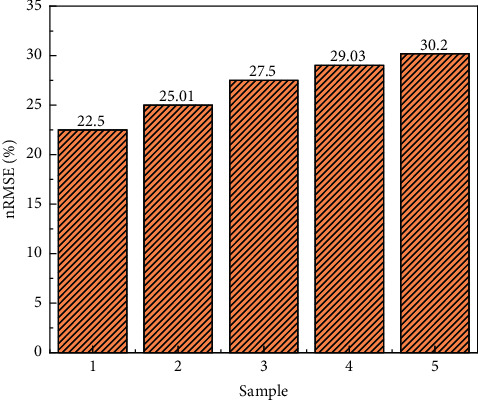
nRMSE of CNN-SSA-Bi-LSTM for predicting every 45 min.

According to the normal course teaching, the ultra-short-term forecast is a week of teaching time, and the predicted value of the teaching effect every 45 minutes, with a total of 5 forecast points.  Figure 4 shows that CNN-SSA-Bi-LSTM uses the above 4 variables as model input, the step size is 6, and it predicts the teaching method of the experimental data for one week, takes a predicted value every 45 minutes, and calculates the nRMSE of the model at each predicted value point.  Figure 3 shows that as the prediction time interval becomes larger, the prediction effect of the model gradually deteriorates. The first 45-minute prediction effect is the best, and the fifth 45-minute prediction performance is the worst, and its nRMSE is as high as 30.2%. This is because the classroom effect is better. With the increase of courses, the longer it takes to digest, the average effect increases slowly. The nRMSE calculated *i* is relative to the average of all target values, which is different from the maximum value of the target value in the test set divided by the partially published literature.

In summary, the poor prediction results of the LSTM model may be attributed to the fact that the model belongs to the “black box model,” which is directly predicted based on a single time series, while the normal teaching effect has many influencing factors, and the influencing mechanism is more complicated. As a result, the prediction accuracy of a single time series model is limited, and even the prediction results cannot meet the actual needs. The CNN-SSA-Bi-LSTM model can achieve high prediction accuracy in both the training period and the verification period. The CNN model can denoise the data sequence and extract the complex and effective information hidden in the data, it can better reflect the internal mechanism that affects flipped classroom teaching, and SSA optimizes the optimal model parameters of LSTM and improves the efficiency of model parameter selection. In addition, Bi-LSTM has higher prediction accuracy than LSTM. It shows that the CNN-SSA-Bi-LSTM model is feasible for monthly runoff prediction.

## 4. Conclusion

This paper proposes an ultra-short-term prediction model based on CNN-SSA-Bi-LSTM for flipped classroom hybrid teaching research. The model uses one-dimensional convolutional neural network fusion to extract 4 variable data, takes advantage of the two-way long short-term memory network to process time series problems, and combines the Dropout layer and the fully connected layer to achieve ultra-short-term prediction of classroom effect 45 minutes in advance. Through multiple sets of experiments, the rationality and prediction performance advantages of the proposed CNN-SSA-Bi-LSTM model are verified. The following conclusions are obtained: (1) compared with the continuous model, the proposed model increased the relative variance by about 16.22% in the GHI prediction 45 minutes in advance, which is better than many other comparative models; (2) all four variables discussed in this paper help to improve prediction performance, with the student grouping of historical measurements having the greatest impact on the predictive performance of the proposed model. When it is missing as input, the model predicts the worst, with a predictor nRMSE of 24.1%; (3) when making ultra-short-term forecasts, the longer the forecast interval, the worse the model prediction performance. When predicting the classroom effect on the fifth day in the future, the nRMSE value of the proposed model is 30.2%.

## Figures and Tables

**Table 1:** Performance statistics of different models for predicting classroom teaching.

Model	*R*	nMBE (%)	nMAE (%)	nRMSE (%)
SVM	0.93	0.60	19.05	26.15
BPNN	0.92	0.52	15.48	25.11
ELM	0.93	−0.15	14.75	24.90
LSTM	0.94	3.04	14.41	24.34

**Table 2:** Statistical table of performance predicted by different LSTM models.

Model	*R*	nMBE (%)	nMAE (%)	nRMSE (%)
Continuous model	0.93	1.01	15.50	25.51
Univariate LSTM	0.94	3.02	14.41	24.34
Multivariate LSTM	0.94	−1.86	13.40	23.27
Multivariate Bi-LSTM	0.94	−1.31	13.21	23.14
Multivariate CNN-Bi-LSTM	0.95	−0.31	12.39	21.96
Multivariate CNN-SSA-Bi-LSTM	0.96	−0.18	11.28	20.09

## Data Availability

The dataset can be accessed upon request.

## References

[1] Chapelle C. A. (2001). *Computer Application in Second Language Acquisition: Computer-Assisted Language Testing*.

[2] Feng M. (2021). Research on the construction of student ability evaluation system based on computer application. *Journal of Physics: Conference Series*.

[3] Zhu Q., Kumar P. M., Alazab M. (2021). Computer application in game map path-finding based on fuzzy logic dynamic hierarchical ant colony algorithm. *International Journal of Fuzzy Systems*.

[4] Wang J., Liu B. J., He W., Xue J. K., Han X. Y. (2021). Research on computer application software monitoring data processing technology based on NLP. *IOP Conference Series: Materials Science and Engineering*.

[5] Bemer M., Grossniklaus U. (2012). Dynamic regulation of Polycomb group activity during plant development. *Current Opinion in Plant Biology*.

[6] Ibrahim M., Muralidharan S., Deng Z., Vahdat A., Mori G. A hierarchical deep temporal model for group Activity recognition.

[7] Oyama H., Watanabe N., Ono Y., Sakashita T. (2008). Community-based suicide prevention through group activity for the elderly successfully reduced the high suicide rate for females. *Seishin shinkeigaku zasshi = Psychiatria et neurologia Japonica*.

[8] Mittermeier R. A. (1973). Group activity and population dynamics of the howler monkey on Barro Colorado Island. *Primates*.

[9] Ellis R. A., Steed A. F., Applebee A. C. (2006). Teacher conceptions of blended learning, blended teaching and associations with approaches to design. *Australasian Journal of Educational Technology*.

[10] Gagnon M. P., Gagnon J., Desmartis M., Njoya M. (2013). The impact of blended teaching on knowledge, satisfaction, and self-directed learning in nursing undergraduates: a randomized, controlled trial. *Nursing Education Perspectives*.

[11] Sutskever I., Hinton G. E., Taylor G. W. (2008). *The Recurrent Temporal Restricted Boltzmann Machine*.

[12] Cronjé J. C. (2011). Using Hofstede’s cultural dimensions to interpret cross-cultural blended teaching and learning. *Computers & Education*.

[13] Ma M. Multimedia Emergency Event Extraction and Modeling Based on Object Detection and Bi-LSTM Network.

[14] Wan X., Wan J., Xu M. (2021). Enteromorpha coverage information extraction by 1D-CNN and Bi-LSTM networks considering sample balance from GOCI images. *Ieee Journal of Selected Topics in Applied Earth Observations and Remote Sensing*.

[15] Du L., Cao D., Li J., Ye H. (2021). Classification and extraction of medical clinical trial screening standard texts based on Bi-LSTM and Attention mechanism. *IOP Conference Series: Earth and Environmental Science*.

[16] Shao S., Wang T., Song C., Su Y., Wang Y., Yao C. (2021). Fine-grained and multi-scale motif features for cross-subject mental workload assessment using Bi-lstm. *Journal of Mechanics in Medicine and Biology*.

[17] Xueqing Z., Zhansong Z., Chaomo Z. (2021). Bi-LSTM deep neural network reservoir classification model based on the innovative input of logging curve response sequences. *IEEE Access*.

[18] Ren S., He K., Girshick R., Sun J. (2017). Faster R-CNN: towards real-time object detection with region proposal networks. *IEEE Transactions on Pattern Analysis and Machine Intelligence*.

[19] Tzoumpas K., Estrada A., Miraglio P. (2022). *A Data Filling Methodology for Time Series Based on CNN and (Bi)LSTM Neural Networks*.

[20] Wikipedia F., Encyclopedia T. F. (2014). *Bayesian Information Criterion*.

[21] Roska T., Chua L. O. (1993). The CNN universal machine: an analogic array computer. *IEEE Transactions on Circuits and Systems II: Analog and Digital Signal Processing*.

[22] Roska T., Chua L. O. (2008). The CNN universal machine. *Journal of Circuits, Systems, and Computers*.

[23] Gers  F. A., Schmidhuber J., Cummins F. (2000). Learning to Forget: Continual Prediction with LSTM, Neural Computation.

[24] Ma X., Hovy E. (2016). *End-to-end Sequence Labeling via Bi-directional LSTM-CNNs-CRF*.

[25] Gers F. A., Schmidhuber E. (2001). LSTM recurrent networks learn simple context-free and context-sensitive languages. *IEEE Transactions on Neural Networks*.

[26] Nema S., Goulermas J. Y., Sparrow G., Helman P. (2011). A hybrid cooperative search algorithm for constrained optimization. *Structural and Multidisciplinary Optimization*.

[27] Song C., Yao L., Hua C., Ni Q. (2021). Comprehensive water quality evaluation based on kernel extreme learning machine optimized with the sparrow search algorithm in Luoyang River Basin, China. *Environmental Earth Sciences*.

[28] Özer İ., Efe S. B., Özbay H. (2021). CNN/Bi‐LSTM‐based deep learning algorithm for classification of power quality disturbances by using spectrogram images. *International Transactions on Electrical Energy Systems*.

[29] Chen H., Wu W., Liu H. B. (2015). Assessing the relative importance of climate variables to rice yield variation using support vector machines. *Theoretical and Applied Climatology*.

[30] Hannah Inbarani H., Nizar Banu P. K., Azar A. T. (2014). Feature selection using swarm-based relative reduct technique for fetal heart rate. *Neural Computing & Applications*.

